# Toward early screening for early management of postnatal depression? Relationships between clinical signs present in the infant and underlying maternal postnatal depression

**DOI:** 10.3389/fpsyt.2022.986796

**Published:** 2022-09-09

**Authors:** Sabrina Julien-Sweerts, Sandie Rousselin, Florence Raffeneau, Charlotte Xavier-David, Violette Changeur, Gisèle Apter, Lucia Romo, Ludovic Gicquel

**Affiliations:** ^1^Université de Reims Champagne Ardenne, C2S EA 6291, Reims, France; ^2^Child and Adolescent Psychiatry Department, Laborit Hospital, Poitiers, France; ^3^Service Universitaire de Pédopsychiatrie du Groupe Hospitalier du Havre, Université Rouen Normandie, Mont-Saint-Aignan, France; ^4^EA 4430 Clipsyd, Paris Nanterre University, Nanterre, France; ^5^Hôpital Universitaire Raymond Poincaré, CESP, U1018 INSERM UPS UVSQ, Garches, France

**Keywords:** screening, maternal postnatal depression, psychopathology, early clinical signs, management

## Abstract

**Objective:**

The objective was to screen for maternal postnatal depression (MPD) by administering the Edinburgh Postnatal Depression Scale (EPDS) during the first “peak” of incidence of MPD (i. e., between the 6th and the 10th week of the infant's life) and to therefore explore the relationship between mothers' EPDS scores and early clinical signs in the infant. We wanted to evaluate the relevance of a diagnostic tool that combines the EPDS with questions focused on clinical signs displayed by the infant.

**Participants:**

Seven hundred and sixty seven mothers aged 18–46 (*M* = 30.5, *SD* = 4.9) participated in the study, representing 49.2% of all women who delivered in the study area during the research inclusion period. Main outcome measures: Sociodemographic data were collected. MPD was measured by EPDS (score ≥ 12). The presence of clinical signs in the infant was investigated by closed (i.e., yes or no) questions inquiring into whether the infant has or has had difficulty sleeping, feeding difficulties, crying difficult to calm, or other difficulties.

**Results:**

The prevalence of MPD in our sample was 22.16%. The relationships between MPD and early clinical signs present in the infant, i.e., sleep difficulties, feeding problems, crying difficult to calm (*p* < 0.001), and other problems (*p* = 0.004), were very significant, as confirmed by a chi-square test of independence. In particular, sleep difficulties (OR = 2.05, CI 1.41–2.99) and feeding difficulties (OR = 1.59, CI 1.10–2.30) seemed to predict MPD.

**Conclusions:**

Early clinical signs in the infant can alert the medical team to potential psychological suffering on the part of the mother, at which time the EPDS can be proposed. The use of this method has the potential to improve screening for, and therefore early management of, MPD.

## Background and objectives

The postpartum period is particularly vulnerable to decompensation and even the outbreak of psychiatric pathologies, including depression (1). Perinatal depression is defined in the Diagnostic and Statistical Manual of Mental Disorders, Fifth Edition (DSM-5) as an episode of major depressive disorder (MDD) with peripartum onset, i.e., symptom onset during pregnancy or in the 4 weeks following delivery (2).

The prevalence of maternal perinatal depression (MPD) is currently considered to be 10–20% (3, 4). A recent systematic review and meta-regression suggested that the overall pooled prevalence of perinatal depression is 11.9% (95% confidence interval [CI], 11.4%−12.5%) (5). In France, it is between 5 and 20%, depending on the diagnostic criteria and measurement tools used (6).

Both biological factors (e.g., sex hormones, stress hormones, thyroid hormones) and psychosocial factors contribute to the development of postpartum depression (7). Lower socio-economic status, lower educational level, younger age, not being married, lack of support and empathy from partner, lack of social support, perceived stress, prior history of depression, current depressive symptoms, and a history of sexual or physical violence are frequently identified as potential risk factors for perinatal depression (8–11). The biopsychosocial model of MPD, as supported by several meta-analyses, attempts to comprehensively understand the risk factors for the onset of depressive symptoms as well as the protective factors associated with this disorder, with the aim of improving prevention and treatment (12–14).

In fact, MPD has major consequences for public health. In France, a recent national survey showed that suicide has become the second leading cause of postpartum maternal mortality (accounting for 13.4% of maternal deaths), behind cardiovascular diseases (13.7% of maternal deaths) (15). Untreated MPD seems to have negative consequences for both infants and mothers. A recent systematic review highlighted the adverse effects of MPD on mothers (physical health, psychological health, worse quality of life, difficulties in social relationships, and risky behaviors such as substance abuse), on mother-child interactions (attachment difficulties, insecure attachments), and on infants (anthropometry; physical health; sleep; and motor, cognitive, language, emotional, social, and behavioral development) (16). Early functional signs such as diarrhea (17, 18), constipation, colic (19), gastroesophageal reflux (20), night-time awakening, and other infant sleep troubles (21, 22) seem to be more frequent in infants with a mother suffering from MPD. Difficulty with breastfeeding has also been connected to postnatal depression (23). In general, maternal depression has been associated with excessive crying and feeding and sleeping problems in infants (24).

However, screening and diagnosis present massive difficulties. As many as half of the mothers suffering from maternal postnatal depression would not be spotted by their close circle (her family, friends, doctors) (25). In light of the potential consequences, early screening for the risk of postnatal depression seems essential.

The Edinburgh Postnatal Depression Scale (EPDS) is one of the most widely used screening instruments for assessing symptoms of maternal postnatal depression (26, 27). The main objective of the present study was to assess the prevalence of MPD in a French area of practice by administering the EPDS during the first “peak” of MPD incidence (i.e., between the 6th and the 10th week of the infant's life) and to concomitantly screen for early clinical signs in the infant. The objective was to explore the relationship between mothers' sociodemographic characteristics and EPDS scores and any early clinical signs present in the infant, for the purpose of evaluating a potential screening tool for use in the general population that would combine the EPDS with questions focused on functional signs in the infant. In other words, could early clinical signs in the infant signal possible psychological suffering on the part of the mother—and therefore offer an avenue for improving the detection of MPD and beginning treatment as early as possible?

## Methods

### Participants

Women who delivered between 18 December 18 2017 and 30 April 30 2018 and who visited the Vienne PMI (a free public health service offering consultations for children from 0 to 6 years old) were contacted to participate in the study. The inclusion criteria were: mothers having given birth, having an infant aged between 6 and 10 weeks, living in Vienne (a department in eastern France), having internet access, and having given informed consent for the study. We chose “having an infant aged between 6 and 10 weeks” as a criterion because many researchers consider the DSM-5's definition of “postpartum period” (i.e., 4 weeks) to be too short, given what is already well-established in professional practice and in scientific research on the subject (4). The best estimates of the point prevalence of major and minor disorders are in the first three months postpartum. Regarding major depression alone, there are three seemingly “peak” periods of incidence: during the second trimester of pregnancy, 2 months postpartum, and 6 months postpartum (28). The first peak of MPD incidence would therefore be between the 6th and the 10th week of the infant's life (29). The exclusion criterion was low French language or literacy skills, and the outcome of the study was infant death.

### Measures

Sociodemographic data were collected. MPD was measured by the French version of the EPDS, a validated 10-item self-report questionnaire that can be completed in just a few minutes (30, 31). The EPDS can detects depression in mothers from the 4th week postpartum (31) by assessing emotional experiences over the past 7 days (26). Mothers respond on a Likert scale between 0 (“not at all”) and 3 (“yes, most of the time” or “quite a lot”). The French version has good psychometric properties, and item comprehension is excellent and unequivocal (30).

The best cutoff value for the EPDS to screen for depression, according to both the DSM-5 and the International Classification of Diseases, Tenth Revision (ICD-10), would be 11 or higher (32), as this value maximizes the combined sensitivity and specificity (81 and 88%, respectively) (27). In the French study, a cutoff score of 11.5 for screening for major depressive disorder provided optimum results, with 80% sensitivity and specificity (30). For the present study, the EPDS cutoff value was 12.

Finally, the presence of early clinical signs in the infant was investigated via the use of closed (i.e., yes or no) questions, that asked whether the infant has or has had difficulty sleeping, feeding difficulties, crying difficult to calm, or other difficulties identified by the mother. Mothers were also able to add a comment to express themselves freely on points not addressed or to clarify a response.

### Data analysis

The statistical analyses were carried out using Jamovi, version 1.0.8.0. The present study first examined postpartum depression based on the EPDS score, a quantitative variable. The Pearson correlation test was used to study the relationship between this variable and age (a quantitative variable). The study then focused on EPDS score as a qualitative variable (negative if EPDS <12 and positive if ≥ 12, our threshold score for postpartum depression). The relationship between the EPDS qualitative variable and other qualitative variables (e.g., parity, marital status, occupational status, presence of early functional signs in the infant) was studied using the chi-square test of independence. Finally, a multivariate analysis of the qualitative variable EPDS was carried out.

## Results

### Sample

Nearly half (49.2%) of all women who delivered in the study area during the research period participated in the study, representing a total of 767 mothers aged between 18 and 46 (*M* = 30.5, *SD* = 4.9), 40.38% of whom were primiparous. In terms of their relationship status, 3.65% were single, 33.12% were in partnered relationships, 2.35% were in partnered relationships living separately, and 60.89% were married couples. Concerning their professional activities, 6.13% had resumed their activity at the time of the survey, 77.44% were on maternity leave, 2.61% were on parental leave, 0.91% were students, and 12.91% were unemployed.

### EPDS

The average EPDS score of our sample was 7.42, with a standard deviation of 4.98; the minimum score was 0 and the maximum was 25 (*n* = 767).

In the present study, 22.16% of mothers (*n* = 170) had EPDS scores ≥12, 77.84% (*n* = 597) had scores <12, 25.13% (*n* =193) had scores >10, and 9.5% (n=73) had scores >14.

Age and MPD status were not correlated [*r* (765) = −0.008, *p* = 0.83].

### MPD and qualitative variables

Table 1 summarizes the results between the MPD status and the other qualitative variables.

**Table 1:** Association between MPD status and other qualitative variables.

	**Variable**		**No MPD % (*n*)**	**MPD % (*n*)**	***n***	***N***	**X^2^ statistic^a^ (df)**	***p*-value^a^**
Primiparous	No		77.8 (337)	22.2 (96)	433	766	2.87^−4^ (1)	0.986 ns
	Yes		77.8 (259)	22.2 (74)	333			
Marital status	Single		57.1 (16)	42.9 (12)	28	767	13.1 (3)	0.004**
	Partnered, with common housing		78.3 (199)	21.7 (55)	254			
	Partnered, with separate accommodation		55.6 (10)	44.4 (8)	18			
	Married		79.7 (372)	20.3 (95)	467			
Professional status	Active or student		85.2 (46)	14.8 (8)	54	767	8.90 (3)	0.031*
	Maternity leave		79.1 (470)	20.9 (124)	594			
	Parental leave		70 (14)	30 (6)	20			
	Unemployed		67.7 (67)	32.3 (32)	99			
Early functional signs in the infant	Difficulties with sleeping	no	83.6 (399)	16.4 (78)	477	763	25.8 (1) OR = 2.43	<0.001***
		yes	67.8 (194)	32.2 (92)	286			
	Feeding problems	no	82.1 (384)	17.9 (84)	468	765	12.7 (1) OR = 1.86	<0.001***
		yes	71 (211)	29 (86)	297			
	Crying difficult to calm	no	82.5 (340)	17.5 (72)	412	765	11.6 (1) OR = 1.81	<0.001***
		yes	72.2 (255)	27.8 (98)	353			
	Other problems	no	80.1 (472)	19.9 (117)	589	758	8.10 (1) OR = 1.74	0.004**
		yes	69.8 (118)	30.2 (51)	169			

^a^denotes the use of a chi-square test for independence. *Statistically significant at *a* = 0.05. **Statistically significant at *a* = 0.01. ***Statistically significant at *a* = 0.05.

### MPD and primiparous status

**Figure 1:**
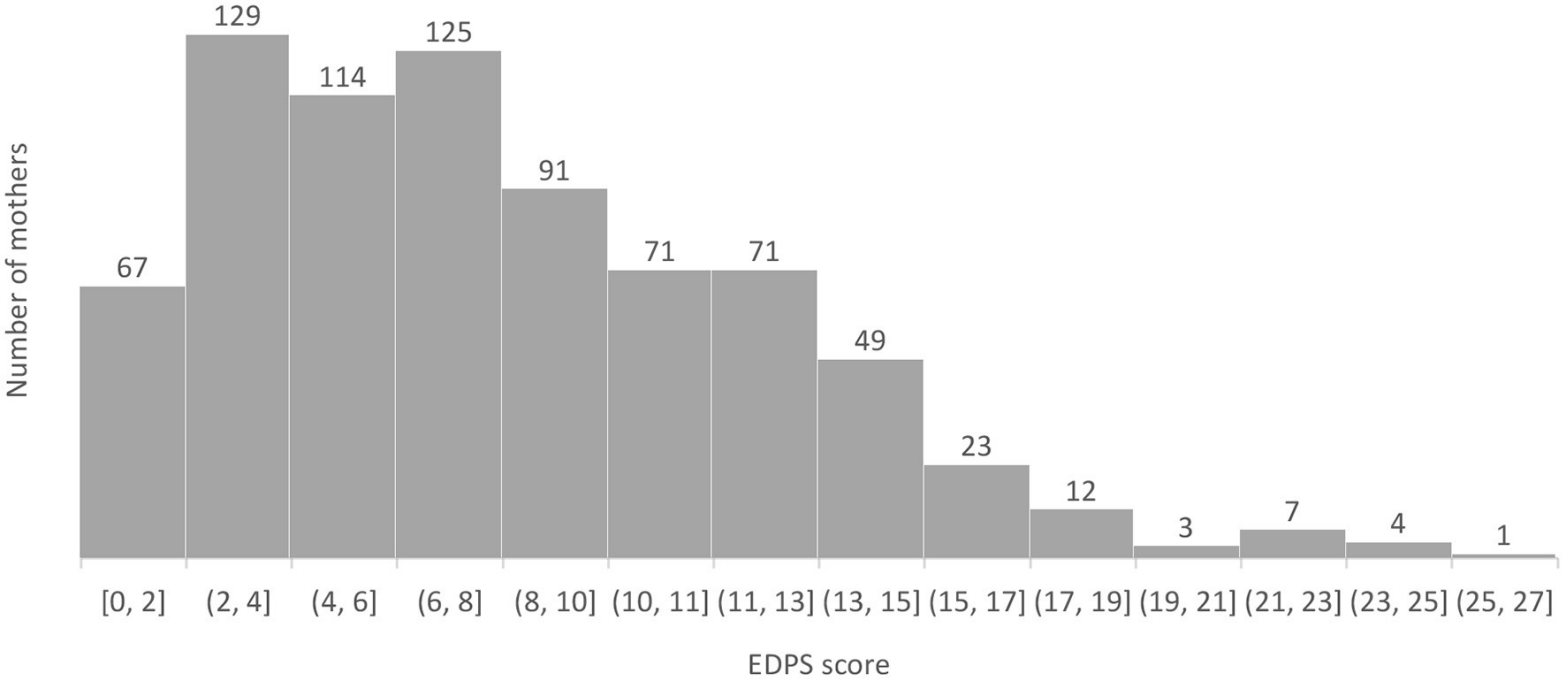
Distribution of EPDS scores.

Figure 1 shows the distribution of EPDS scores. There was no significant relationship between MPD status and being primiparous. A chi-square test of independence was performed to examine the relationship between MPD and being primiparous. The relationship between these variables was not significant.

### MPD and marital status

We detected a significant relationship between these variables. In this study, single mothers or Mothers in couples with separate housing seemed to have an increased risk of MPD. More precisely, 42.9% of single mothers and 44.4% of single mothers of their spouse in our sample met the criteria for MPD.

### MPD and professional status

The relationship between these variables was also significant (We therefore performed a binomial logistic regression to investigate further). Professional status explained only 1% of the MPD observed in the present study. However, the risk of MPD was 2.75 times greater for unemployed women than for women who were students or actively employed.

### MPD and early functional signs in the infant

The relationship between sleep difficulties in the infant and MPD in the mother was very significant. In the present study, 54.1% of infants whose mothers suffered from MPD had sleep problems, compared to 32.7% of infants whose mothers did not suffer from MPD. The risk of having a mother with MPD was 2.43 times greater for an infant suffering from sleep difficulties than for an infant with no sleep difficulties (*OR* [odds ratio] = 2.43).

The relationship between feeding problems in the infant and MPD in the mother was also significant. In the present study, 50.6% of infants whose mothers suffered from MPD had feeding problems, compared to 35.5% of infants whose mothers did not suffer from MPD (*OR* = 1.86).

The relationship between infants experiencing crying that was difficult to calm and mothers experiencing MPD was significant (*OR* = 1.81).

The relationship between infants experiencing other types of distress and mothers experiencing MPD was significant (*OR* = 1.74).

In the binomial logistic regression, we combined the presence of early functional signs in the infant (i.e., sleep or feeding difficulties, crying difficult to calm) with mothers' marital and professional statuses. This model explained only 6.67% of the observed variance in MPD (*pseudo R*^2^ = 0.067). Mothers' marital status, especially the status of single vs. married (*OR* = 2.43), along with the presence of sleep difficulties (*OR* = 2.05) or feeding difficulties (*OR* = 1.59) in the infant, seemed to be the best predictors of MPD. Table 2 summarizes the results of the logistic regression.

**Table 2:** Predictors of MPD.

**Predictors**		**Estimate**	**SE**	**Z**	***p*-value**	**Odds ratio [95% CI]**
Intercept		0.17	0.56	0.30	0.762 ns	1.18 [0.40–3.52]
Marital status	In couple, with common housing, vs. single ^a^	0.84	0.44	1.90	0.058 ns	2.31 [0.97–5.49]
	In couple, with separate housing, vs. single ^a^	−0.25	0.63	−0.39	0.693 ns	0.78 [0.22–2.70]
	Married vs. single ^a^	0.89	0.44	2.05	0.041*	2.43 [1.04–5.67]
Professional status	Maternity leave vs. active or student ^a^	−0.48	0.42	−1.14	0.256 ns	0.62 [0.27–1.41]
	Parental leave vs. active or student ^a^	−0.86	0.65	−1.33	0.182 ns	0.42 [0.12–1.50]
	Unemployed vs. active or student ^a^	−1.04	0.46	−2.26	0.024*	0.36 [0.14–0.87]
Early functional signs in the infant	Sleep difficulties, no vs. yes ^a^	0.72	0.19	3.75	<0.001***	2.05 [1.41–2.99]
	Feeding problems, no vs. yes ^a^	0.46	0.19	2.47	0.013*	1.59 [1.10–3.31]
	Crying difficult to calm, no vs. yes ^a^	0.30	0.19	1.54	0.124 ns	1.35 [0.92–1.96]

The estimate represents the log odds of EPDS “NO” vs. “YES”; CI, confidence interval; ^a^ = reference category. *Statistically significant at *a* = 0.05. **Statistically significant at *a* = 0.01. ***Statistically significant at *a* = 0.05.

## Discussion

The prevalence of MPD in our sample, 22.16%, is consistent with the literature, although rather high. However, with an EPDS cutoff value of 12, it is unlikely that we have overestimated the prevalence. Indeed, according to a recent review, the mean EPDS cutoff score used in the literature is 11.5 (range 10 to 14), while the most frequently used score is 10^4^. Concerns about overestimation could arise from the fact that the EPDS, the tool most often used in studies, also captures adjustment disorder, a stress-related, time-limited, and non-psychotic disturbance that features anxiety, depressed mood, and a feeling of inability to cope and plan ahead. Adjustment disorder is seen as an understandable but maladaptive response to a stressful event, in this context the birth of an infant, that resolves spontaneously when the stressor is removed or a new level of adaptation is reached (33). In all cases, the mother suffers from a mental disorder and her infant feels it. Yet, the mother's mental health during pregnancy and the first year postpartum is very important for the cognitive, social, and emotional development of her child (34). It should be noted that the data were collected before the COVID-19 pandemic. It is very likely that the prevalence is even higher today because the pandemic has had a significant impact on the mental health of mothers (35).

In our study, and in accordance with the literature, being a single mother or being part of a couple that does not share accommodation was positively associated with the risk of MPD. This variable can even be the most highly predictive of MPD, with one study finding an OR of 3.06 for the latter (36). As of 2018, 59.3% of French women were legally single, divorced, or widowed (37) yet these risk categories would concern “only” 8.4% of mothers. More recently, the COVID-19 pandemic has increased the lack of social support (via measures such as lockdowns, isolation, and social distancing) as well as the risk of depression among vulnerable populations, such as pregnant women (38). It therefore may be appropriate to provide home-based care for these single mothers.

Being unemployed appears to be associated with an increased risk of MPD (OR = 0.36), but being on maternity or parental leave does not. This suggests that is not the fact of not working that increases the risk of MPD, but rather the professional uncertainty and/or financial precarity that accompany being unemployed. Indeed, lower socioeconomic status and lack of financial support have already been identified as risk factors in several studies (8).

We also found an association between sleep or feeding difficulties in the infant and MPD in the mother. According to the literature, 15% to 35% of parents observe sleep difficulties in their child during the first 6 months of life (39). The high percentage we observed can be explained by our methodology, namely, a question linked to the subjective interpretation of the mother without defined criteria. In fact, 37.4% of mothers in our study reported that their infants had difficulty sleeping: 32.7% in the non-MPD group and 54.1% in the MPD group. Newborn babies of depressed mothers have more sleep disorders, including decreased durations of deep sleep and increased durations of disorganized sleep (40). If the sleeping difficulties experienced by infants are correlated with MPD in mothers, perhaps this sign should alert practitioners to screen for MPD in the mother. This is especially important because a baby's sleep problems disrupt the parents' sleep, which can lead to parental fatigue, reduced ability to care for the baby effectively, and symptoms of postpartum depression (41). Taking care of these mothers as early as possible is therefore essential for them and their baby.

In our study, feeding difficulties included colic, regurgitation, gastroesophageal reflux, difficulties in breastfeeding or bottle feeding, and difficulties with weight gain. Feeding difficulties were observed by 35.5% of mothers in the non-MPD group and 50.6% of mothers in the MPD group, and these results are concordant with the literature. Breastfeeding difficulties and concerns about infant weight gain are predictors of maternal distress (36). In addition, colic in infants is associated with a higher risk of depression in the mother, with odds ratios between 2 (95% CI 1.1–3.7) (42) and 3.7 (95% CI 1.4–10.1) (43). The presence of colic is associated with a higher depression score in mothers at 2 months, and this can extend up to 4 months later even if the colic resolves (43). The identification of feeding problems in infants should therefore alert the pediatrician to potential psychological distress in the mother, as this can allow for early management of MPD.

### Limitation

Our study has some limitations. The prevalence in our study is an estimation conducted at the moment of the first “peak” of MPD (between the 6th and the 10th week of the infant's life). It is also plausible that, in recruiting the most available participants for the study, we excluded women with more significant symptoms. The figure of 22.16% therefore may not reflect the complete prevalence. Moreover, our study does not provide information on the mothers' experiences with depression and/or anxiety before and during pregnancy. Another study that conducted a longitudinal investigation using structural equation modeling found that prenatal depressive symptoms were the greatest predictor of postpartum depression (44). It is therefore likely that several mothers among the 22.16% experienced depression before or during the pregnancy that continued afterwards, in line with the findings of several other studies (45, 46). This highlights the relevance of the concept of perinatal depression for the prevention and screening of this disorder. Moreover, even as the analyses carried out here allow us to talk about infants' clinical signs as potential predictors of postnatal depression, it is obvious that postnatal depression in the mother could also be considered a predictor of clinical signs found in the infant. Nevertheless, whatever the predictors used, the presence of one allows us to look for the presence of the other due to their correlation.

### Perspectives

Routine screening is strongly encouraged by the American College of Obstetricians and Gynecologists (ACOG), the US Preventive Services Task Force, and the National Collaborating Centre for Mental Health in the UK (47). ACOG recommends early postpartum follow-up care, including screening for depression and anxiety, for all postpartum women (8). In France, a national survey on maternal mortality highlighted that maternal suicides are not only one of the two leading causes of maternal death, but also one of the most preventable (15). Experts recommend screening for psychological disorders, to identify the warning signs, to understand the indications for psychiatric hospitalization, to train and raise awareness of mental health in perinatal care, and to develop resources within the geographic territory. The use of the EPDS, a very rapid tool recommended many countries' postpartum programs in the primary health care setting, could facilitate the following of these recommendations (48). Pediatricians observing functional signs such as sleep or feeding difficulties in the infant could propose this 10-item questionnaire to the mother.

## Conclusion

MPD is a common disorder, affecting more than one in five mothers. It requires early management to prevent negative consequences for both infants and mothers. Single women or women who live separately from their partners are at higher risk for developing MPD than mothers who are married or live under the same roof as their partners. In addition, clinical signs in the infant such as difficulties with sleeping or feeding are associated with a higher risk of depression in the mother. Their presence should alert the pediatrician to potential psychological suffering on the part of the mother. The EPDS, a 10-item rapid self-assessment scale, should then be used as a first-line tool for this screening.

## Data availability statement

The raw data supporting the conclusions of this article will be made available by the authors, without undue reservation.

## Ethics statement

The studies involving human participants were reviewed and approved by Ethics Committee of the Henri Laborit Hospital, 370 avenue Jacques Coeur, 86 021 POITIERS CEDEX. The patients/participants provided their written informed consent to participate in this study.

## Author contributions

SR designed the study and wrote the protocol. SJ-S performed the main data analysis and wrote the first draft of the manuscript. FR, CX-D, VC, and LG contributed to the assessments and data collection. SJ-S, GA, LR, and LG contributed to the analysis of the results and to the writing of the manuscript. All authors contributed to this article, have approved the final manuscript, have agreed both to be personally accountable for their own contributions, and to ensure that questions related to the accuracy or integrity of any part of the work, even ones in which the author was not personally involved, are appropriately investigated and resolved, and the resolution documented in the literature.

## Conflict of interest

The authors declare that the research was conducted in the absence of any commercial or financial relationships that could be construed as a potential conflict of interest.

## Publisher's note

All claims expressed in this article are solely those of the authors and do not necessarily represent those of their affiliated organizations, or those of the publisher, the editors and the reviewers. Any product that may be evaluated in this article, or claim that may be made by its manufacturer, is not guaranteed or endorsed by the publisher.
